# Transgenic zebrafish for modeling hepatocellular carcinoma

**DOI:** 10.1002/mco2.29

**Published:** 2020-09-03

**Authors:** Joji Nakayama, Zhiyuan Gong

**Affiliations:** ^1^ Department of Biological Sciences National University of Singapore Singapore

**Keywords:** epidemiology of HCC, genetic alterations in HCC, hepatocellular carcinoma (HCC), therapeutic strategies for HCC, zebrafish

## Abstract

Liver cancer is the third leading cause of cancer‐related deaths throughout the world, and more than 0.6 million people die from liver cancer annually. Therefore, novel therapeutic strategies to eliminate malignant cells from liver cancer patients are urgently needed. Recent advances in high‐throughput genomic technologies have identified de novo candidates for oncogenes and pharmacological targets. However, testing and understanding the mechanism of oncogenic transformation as well as probing the kinetics and therapeutic responses of spontaneous tumors in an intact microenvironment require in vivo examination using genetically modified animal models. The zebrafish (*Danio rerio*) has attracted increasing attention as a new model for studying cancer biology since the organs in the model are strikingly similar to human organs and the model can be genetically modified in a short time and at a low cost. This review summarizes the current knowledge of epidemiological data and genetic alterations in hepatocellular carcinoma (HCC), zebrafish models of HCC, and potential therapeutic strategies for targeting HCC based on knowledge from the models.

## Graphical abstract

Liver tumors that developed in zebrafish are highly analogous to liver tumors in humans, and several transgenic zebrafish lines for modeling hepatocellular carcinoma (HCC) have been established. This review summarizes the current knowledge of epidemiological data and genetic alterations in HCC, zebrafish models of HCC, and potential therapeutic strategies for targeting HCC based on knowledge from the models.

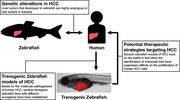

## 1 INTRODUCTION

Liver cancer is the fifth most frequent cancer in the world. Despite aggressive therapy, the 5‐year survival rate of liver cancer patients remains low (below 12% in the United States).^1^ Cancer is the third leading cause of death throughout the world and more than 0.6 million people die from liver cancer annually.^2^ Liver cancer mortality is also due to its resistance to existing anticancer regents; therefore, new therapeutic strategies to eliminate malignant cells from liver cancer patients are urgently needed.

Recent advances in high‐throughput genomic technologies have made it possible to identify de novo oncogene candidates and pharmacological targets through analyzing large numbers of samples from cancer patients. Whole‐genome sequencing analysis of clinical HCC samples has revealed that etiological background influences somatic mutation patterns and the subsequent carcinogenesis of HCC.^3^ Another genomic high‐resolution, copy‐number, and whole‐exon sequencing analysis has identified 135 homozygous deletions and 994 somatic mutations and predicted functional consequences from HCC samples.^4^ However, determining whether identified genetic alterations truly confer malignant transformation of normal cells requires in vivo examinations of genetically modified animals with designed gene mutations that mimic human data. Since the mid‐1980s, a tremendous knowledge of cancer‐related genes has been accumulated by investigating genetically modified mice that either harbored an oncogene or lacked a tumor suppressor gene. These in vivo models have demonstrated transformations of normal cells into malignant cells by both gain‐ or loss‐of‐function cancer‐related genes, thus providing fundamental knowledge about the molecular malignant transformation mechanisms induced by oncogenes, as well as the kinetics and therapeutic responses of spontaneous tumors in intact tumor microenvironments.^5^ The mouse model has several disadvantages despite these findings, including the high cost of animal husbandry, the difficulty of obtaining a large number of lab animals, and the labor intensity and time required to establish and perform in vivo assays, which prevent rapid and high‐throughput studies.

Zebrafish (*Danio rerio*) as a cancer model offers unique advantages.^6^, ^7^, ^8^ For example, (a) zebrafish have orthologues for 86% of 1318 human drug targets, 71% of human proteins, and 82% of disease‐causing human proteins^9^, ^10^; (b) drugs are administered to zebrafish by just dissolving the drugs in water where zebrafish are maintained; (c) the transparency of zebrafish embryos enables to observe directly some processes during tumor development and regression^11^; (d) zebrafish generate large numbers of progeny (100‐200 eggs per one female zebrafish), that is effective in statistical analyses; and (e) cost of maintaining zebrafish is low.

In the past few years, we have been studying liver cancers in zebrafish both via chemical carcinogens and transgenic approaches.^12^, ^13^, ^14^ Comparative transcriptomic analyses have revealed high conservation of molecular signatures and progression between zebrafish and human liver tumors.^15^ In this review, we summarized the epidemiological data and genetic alternations of human liver cancer, the current knowledge from zebrafish HCC analyses, and the potential of developing therapeutic strategies for the treatment of HCC.

## 2 EPIDEMIOLOGICAL STUDIES OF LIVER CANCER

Hepatocellular carcinoma (HCC) is the most common type of adult liver cancer and represents over 90% of all cases of primary liver cancer.^16^ The majority of HCC cases result from infection with chronic hepatitis B virus (HBV) (54%) and hepatitis C virus (HCV) (31%). However, substantial geographic variation in these data exists. In Africa and Asia, more than 60% of HCC patients are HBV carriers, 20% are HCV carriers, and the remaining 20% cases are resultant from alcohol abuse or dietary exposure to aflatoxin B1. In contrast, in the United States, Europe, Egypt, and Japan, the rate of HCV and HBV carriers in HCC patients is more than 60% and 20%, respectively, and the remaining cases are caused by excessive alcohol consumption or metabolic liver diseases.^17^

Clinical studies have revealed that HCC harbors a large number of clonally integrated HBV genomes and that the incidence of HCC development is proportional to serum HBV DNA level.^18^, ^19^, ^20^ Experimental studies in transgenic mice expressing the HBV component protein demonstrated that HCC developed spontaneously.^21^, ^22^, ^23^ Clinical studies indicate a strong association between HCV infection and HCC development.^24^ Experimentally, transgenic mice expressing HCV core proteins also develop HCC.^25^

Alcohol consumption is an avoidable risk factor for HCC development. Clinical studies have revealed that the risk leading to HCC development increases when daily alcohol consumption exceeds 80 grams per day for more than 10 years.^26^ Ethanol is absorbed by the small intestine and metabolized mainly in the liver. Alcohol dehydrogenase oxidizes ethanol to acetaldehyde, which is converted into acetate by aldehyde dehydrogenase. When alcohol consumption is high, cytochrome P450 2E1 (CYP2E1) also catalyzes ethanol into acetaldehyde but produces reactive oxygen species (ROS). Acetaldehyde and ROS function as carcinogens and interfere with DNA synthesis and repair.^27^

Dietary exposure to aflatoxin B1 is another major risk factor for HCC development. Aflatoxin B1 is a genotoxic hepatocarcinogen that causes cellular transformation by inducing DNA adducts, leading to genetic alternations in target cells. Aflatoxin B1 is metabolized by cytochrome‐P450 enzymes into aflatoxin B1‐8, 9‐epoxide, a reactive intermediate, which then binds to genomic DNA in liver cells and results in DNA adducts.^28^ In geographic areas with high exposure to dietary aflatoxin B1, such as China and Africa, a transversion of guanine to thymine at the third position of codon 249 serine in *TP53* is often observed.^29^, ^30^, ^31^ Experimental studies by exposing human HCC cell lines to aflatoxin B1 have also induced similar transversion in codon 249 of *TP53*.^32^, ^33^

## 3 MOLECULAR PATHOGENESIS OF HCC

Studies over the past four decades have demonstrated that cancer cells develop by accumulating numerous genetic mutations, which leads to the overexpression of oncogenes and/or loss of tumor suppressor genes; these mutations confer proliferation, survival, and drug resistance to cancer cells.^34^, ^35^ These mutations are also observed in the development and progression of HCC,^36^, ^37^ as summarized in Table 1. This section describes the genetic alternations that are most frequently observed in HCC.

**TABLE 1:** A list of genetic mutations which are observed in HCC

Gene	Function of gene product	Cause of genetic alteration	Frequency	Reference
*APC*	Signaling suppressor	Loss of function mutations of APC gene	3%	^15^
*AXIN1*	Signaling suppressor	Loss of function mutations of AXIN gene	5%	^60^
*AXIN2*	Signaling suppressor	Somatic mutation at codon 688 of AXIN2 gene	2%	^43^
*BRAF*	Kinase	Somatic mutation at codon 600 of B‐raf gene	15%	^130^
*BRCA2*	DNA repair regulator	Chromosomal deletion in 13q	43%	^45^
*C‐MET*	Tyrosine kinase receptor	Overexpression	69%	^131^
*C‐MYC*	Transcriptional factor	Chromosomal amplification in 8q	48%	^95^
*CCND1*	Cyclin‐dependent kinase	Chromosomal amplification in 11q13	13%	^132^
*CDKN2A*	Cyclin‐dependent kinase inhibitor	Decreased expression through promoter methylation	62%	^133^
*CTNNB1*	Transcriptional factor	Somatic mutation	20‐40%	^61^
*EGFR*	G‐protein coupled receptor	Overexpression	53%	^75^
*ERBB3*	G‐protein coupled receptor	Overexpression	63%	^75^
*FLT1*	Tyrosine kinase receptor	Overexpression	50%	^134^
HBV (DNA virus)	Virus proteins	HBV integration	54‐60%	^17^
HCV (RNA virus)	Virus proteins	HCV integration	20‐31%	^17^
*IGF2*	Growth Factor	Elevated expression	14%	^135^
*JAK1*	Kinase	Somatic mutations	9%	^136^
*KDR2*	Tyrosine kinase receptor	Overexpression	86%	^137^
*KRAS*	GTPase	Somatic mutation at codon 12 of k‐ras	3%	^90^
*PDGFRA*	Tyrosine kinase receptor	Overexpression	63%	^138^
*PDGFRB*	Tyrosine kinase receptor	Overexpression	19%	^137^
*PTEN*	Tyrosine phosphatase	Chromosomal deletion in 10q23	44%	^139^
*RB1*	Cell cycle regulator	Chromosomal deletion in 13q	43%	^45^
*SATB1*	Genomic organizer	Ecotopic expression	57%	^140^
*SOCS1*	Signaling suppressor	Decreased expression through promoter methylation	65%	^141^
*SOCS3*	Signaling suppressor	Decreased expression through promoter methylation	33%	^142^
*TGFA*	Growth Factor	Elevated level in urine	65%	^143^
*TP53*	Transcriptional factor	Somatic mutation at codon 249 of p53 gene	23%	^144^
*VEGFA*	Growth Factor	Chromosomal amplification in 6p21	4‐6%	^145^

### 3.1 HBV and HCC

As mentioned above, the leading cause of HCC is HBV. This DNA virus is a noncytopathic, partially double‐stranded, and hepatotropic. The HBV DNA encodes reverse transcriptase/DNA polymerase (pol), the capsid protein known as hepatitis B core antigen, envelope proteins (L, M, and S), and protein X (HBx) (whose functions are not fully understood).^38^

Comparative genomic hybridization and loss of heterozygosity analyses of HCC samples have revealed chromosomal instability in more than 80% of HBV‐associated tumors, including gains of chromosomes 1q, 5, 6q, 7q, 8q, 17q, and 20 and loss of chromosomes 1p, 4q, 6q, 8p, 13q, 16, 17p, and 21.^4^, ^39^, ^40^, ^41^ Representative oncogenes such as *CCNA1* and *Myc* lie on 4q and 8p, respectively, whereas representative tumor suppressor genes such as *RB1*, *TP53*, *AXIN1*, and *CDH1* lie on 13q, 17p, 16p, and 16q, respectively.^42^, ^43^, ^44^, ^45^

Experimental studies have demonstrated that transgenic mice expressing the *HBx* gene specifically succumb to progressive histopathological changes in the liver, initially with multifocal areas of altered hepatocytes, followed by the appearance of benign adenomas, and then the development of malignant carcinomas.^22^ In contrast, transgenic mice producing only HBV L envelope protein uniformly develop HCC.^21^ Other transgenic mice expressing HBsAg develop necroinflammatory liver disease, which progresses into HCC.^23^ These observations suggest that HBV‐driven proteins themselves are sufficient to cause the malignant transformation of hepatic cells. In cooperation with their transformation ability, the integration of HBV DNA into the host genome in hepatic cells accelerates hepatic carcinogenesis by inducing genomic instability in hepatic cells. This results in the elevated expression of oncogenes and decreased expression of tumor suppressor genes.

### 3.2 HCV and HCC

The second leading cause of HCC, HCV, is a completely cytoplasmic replicating RNA virus that maintains its genome as an episome associated with the endoplasmic reticulum. The HCV‐positive stranded RNA genome encodes the core protein, envelope proteins (E1 and E2), and nonstructural proteins (NS2, NS34, NS4A, NS5A, and NS5B) for viral replication, assembly, and maturation. Among these viral components, the HCV core proteins, E1, and E2 induce HCC in transgenic mice by overexpression.^25^, ^38^, ^46^, ^47^

The HCV core protein is reported to suppress the host immune responses. It inhibits Fas/TNF‐α‐mediated apoptosis in HCV‐infected hepatocytes by binding to the cytoplasmic domains of TNFR1 and lymphotoxin b receptor; it suppresses antiviral cytotoxic T‐lymphocyte responses through its interactions with the gC1q receptor.^48^, ^49^, ^50^, ^51^ Interference with the host immune responses results in infected hepatocyte survival, which promotes persistent HCV infection. Although transgenic mice expressing the HCV core protein developed HCC, the transgenic mice did not show impaired immune responses to adenovirus infections.^25^, ^52^ It has also been reported that transgenic mice expressing both E1 and E2 show accelerated diethylnitrosamine (DEN)‐induced tumor formation as a result of suppressed apoptosis.^46^

### 3.3 Wnt/β‐catenin signaling pathway

The Wnt/β‐catenin signaling pathway regulates stem cell pluripotency and cell fate decisions during development.^53^, ^54^ Wnt ligands are secreted glycoproteins that bind to Frizzled receptors and confer β‐catenin transcriptional activity. The dual biological functions of β‐catenin are to mediate Wnt signaling and promote cell‐to‐cell adhesion by binding with E‐cadherin at adherens junction sites. In the absence of Wnt ligands, the cytoplasmic pool of β‐catenin is tightly regulated by proteasomal degradation, a process where β‐catenin is phosphorylated by a complex of casein kinase 1α (CK1α), the scaffold protein AXIN, and the tumor suppressor adenomatous polyposis coli (APC). The phosphorylated β‐catenin is then subjected to ubiquitylation and proteasomal degradation. Binding Wnt to its receptors prevents AXIN and APC from interacting with β‐catenin. This results in β‐catenin stabilization and translocation into the nucleus, where β‐catenin associates with T‐cell factors (TCF) to activate target genes.^53^

Aberrant regulation of Wnt/β‐catenin signaling has been frequently observed in many types of cancers.^55^ Clinical studies conducted by different groups have revealed that somatic mutations of the β‐catenin gene leading to β‐catenin stabilization occurs in 20‐40% of HCC cases.^43^, ^56^, ^57^, ^58^ Genetic alternations that cause constitutive stabilization of β‐catenin, such as through loss of function mutations of APC and AXIN genes, have also been observed in HCC.^59^, ^60^, ^61^ Furthermore, experimental studies have demonstrated that 67% of *APC* knockout mice develop HCC via activated β‐catenin‐mediated transcription.^62^ Transgenic mice expressing an oncogenic mutant form of β‐catenin (a truncated NH2 terminus) also rapidly develop hepatomegaly via inhibiting apoptosis.^63^ Disruption of β‐catenin/Tcf4 complex with β‐catenin/Tcf inhibitors such as PKF118‐310, PKF115‐584, or CGP049090 also interferes with the proliferation of cultured human HCC cells (HepG2 and Huh7) in vitro; it also inhibits tumor growth in vivo in the HepG2 xenograft model by inducing apoptosis as well as decreasing the expression of *Myc* and *CCND1*.^64^ These observations suggest that aberrant regulation of Wnt/β‐catenin signaling undoubtedly contributes to the development of HCC.

### 3.4 TP53

TP53 functions largely as a transcription factor and can trigger a variety of antiproliferative programs by activating or repressing key effector genes. As a cellular gatekeeper, TP53 regulates cell division‐related mechanisms to ensure the fidelity of cell division, cellular senescence (a permanent form of cell cycle arrest), apoptosis or programmed cell death, and autophagy (a catabolic process involving the degradation of the cell's components primarily through the lysosomal machinery). In cell cycle checkpoints, TP53 contributes to both G1 and G2/M phase arrests through inducing the transactivation of p21^waf1/cip1^ (a cyclin‐dependent kinase inhibitor) and interfering with the function of cyclin B1/cdc2 complex, respectively.^65^, ^66^ In cellular senescence, TP53 regulates senescence by transactivating p21^waf1/cip1^ and E2F7.^67^ In apoptosis, the function of TP53 is both transcription dependent and transcription independent. In the former, which takes place in the nucleus, TP53 contributes to apoptosis by transactivating numerous genes such as *Bax*, *PIG3*, *Killer/DR5*, *CD95* (*Fas*), *p53AIP1*, *Prep*, *Noxa*, and *PUMA*.^68^ In the mitochondria, TP53 induces outer membrane permeabilization, thus leading to the release of proapoptotic factors from the mitochondrial intermembrane space. In controlling autophagy, TP53 has a dual function: nuclear TP53 initiates autophagy through transactivating *AMPK*, *PTEN*, *Sestrins*, and the damage‐regulated autophagy modulator (*DRAM*) gene that encodes lysosomal protein,^69^ whereas cytoplasmic TP53 inhibits autophagic flux by an unknown mechanism.

Regulation of TP53 is caused by the disruption of its interaction with MDM2, a negative regulator that mediates the ubiquitin‐mediated degradation of TP53. In response to cellular stress, the amino terminus of TP53 is phosphorylated by various kinases, including ATM, ATR, DNA‐PK, CHK1, and CHK2,^70^ which prevents MDM2 binding and leads to the stabilization of TP53.

It has been well documented that the loss of TP53 function is a common feature of many human cancers.^71^, ^72^ It has been shown from clinical studies that HCC samples derived from cancers attributable to HBV or HCV and/or exposure to aflatoxin B1 frequently exhibit a point mutation at the third position of codon 249 serine in *TP53*.^29^, ^31^ Experimental studies have demonstrated that liver‐specific deletions of *TP53* lead to the formation of HCC.^73^ Moreover, a Cre‐loxP‐based strategy to temporally recover Tp53 expression in Tp53 knockout mice has demonstrated that, without affecting normal tissue, restoration of endogenous Tp53 regressed radiation‐induced lymphomas and sarcomas by inducing apoptosis and cellular senescence, respectively.^36^

These observations suggest that TP53 not only functions as an initial barrier against HCC development but also suppresses tumor growth through inducing a variety of antiproliferative programs.

### 3.5 ErbB receptor tyrosine kinase family

The ErbB receptor family consists of four cell surface receptors: ErbB1/EGFR/HER1, ErbB2/HER2, ErbB3/HER3, and ErbB4/HER4. These are cell membrane receptor tyrosine kinases and are activated by ligand binding (ie, EGF, TGFa, AR, and Epigen) and receptor dimerization. They regulate cell proliferation, migration, differentiation, apoptosis, and cell motility by activating PI3K/Akt, MAPK, and many other signaling pathways. The coding genes are often overexpressed, amplified, or mutated in cancers of the lung, breast, ovarian, bladder, and others.^74^

Immunohistochemistry analysis of clinical samples from 100 HCC patients has shown that EGFR expression is elevated in HCC lesions but not in adjacent nontransformed hepatic cells in 53 cases and ErbB3 overexpression in HCC lesions is observed in 64 cases. Furthermore, the elevated expression of EGFR and ErbB3 correlates with more malignant phenotypes such as high proliferating ability, advanced stage, intrahepatic metastasis, and poor disease‐free survival.^75^ Experimentally, constitutive activation of EGFR‐mediated signaling in transgenic mice and zebrafish has demonstrated that EGFR activation indeed results in HCC and the suppression of the EGFR signaling causes regressed HCC.^14^, ^76^, ^77^ Moreover, the blockade of EGFR signaling with gefitinib EGFR inhibitor also prevents DEN‐induced liver carcinogenesis in rats and inhibits the proliferation of human HCC cell lines Huh‐7 and HepG2 in vitro.^78^, ^79^ Thus, EGFR‐mediated signaling plays a critical role in HCC development and maintenance.

## 4 TRANSGENIC ZEBRAFISH MODELS FOR HCC

According to the current knowledge of molecular pathogenesis of HCC, there is no single dominant molecular pathogenesis underlying all HCCs. Therefore, different therapeutic strategies would be required for treating different HCC subclasses due to different genetic alternations. To devise the strategies, different HCC models will be required to mimic different HCC subclasses. Our early comparative transcriptomic analysis demonstrated that liver tumors that developed in zebrafish as a result of exposure to chemical carcinogens are highly analogous to liver tumors in humans.^15^ Based on the molecular pathogenesis of human HCC, several transgenic zebrafish lines with different oncogenes have been established. These lines are summarized in Table 2. This section summarizes the knowledge obtained from studies of these transgenic zebrafish.

**TABLE 2:** A list of transgenic zebrafish for modeling HCC

Transgene	Promoter	Phenotype	Onset period	Frequency	Ref.
*HBx‐mCherry*	*fabp10*	HBx‐mCherry transgenic zebrafish develops HCC in *TP53−/−* background,	11 mpf	44%	^80^
*HCP*	*fabp10*	HCP transgenic zebrafish develops HCC only in combination with thioacetamide (TAA)	6 weeks after TAA treatment		^83^
Loss of *APC* (*APC+/−*)	Null	*APC+/* zebrafish develops spontaneous liver tumor. DMBA treatment increases the frequency to 70.8%.	More than 15 mpf	17.6%	^85^
*ctnnb1/β‐catenin*	*fabp10*	The level of HCC in the fish was 78% and 80% at 6 and 12 mpf, respectively.	6 mpf	78%	^86^
*CTNNB1mt and tcf7l2*	*fabp10*	The double transgenic larvae shows significant hepatomegaly within 3 days from the transgene expression.	Unknown	Unknown	^87^
*Xmrk*	*fabp10*	All of *Xmrk* transgenic zebrafish develops HCC within 3 weeks from the transgene expression.	3 weeks from the transgene induction	100%	^14^
*EGFP‐KrasG12V*	*fabp10*	All of *EGFP‐KRASG12V* transgenic zebrafish develops robust and homogeneous tumors in the liver after 1 month of the transgene induction	1 month from the transgene induction	100%	^93^
*EGFP‐myca*	*fabp10*	5% of EGFP‐myca transgenic zebrafish develops multinodular HCC with cirrhosis at 8‐9 months from the transgene induction.	6‐7 months from the transgene induction	51%	^96^
*EDN1*	*fabp10*	83% of *EDN1* transgenic zebrafish shows steatosis by 5 months, 17‐18% of the fish developed hyperplasia by 7‐9 months, and 17‐20% of the fish exhibited HCC by 11mpf.	7‐9 mpf	17–20%	^101^
*Tgfb1a*	*fabp10*	*Tgfb1a* transgenic zebrafish develops hyperplasia and HCC.	6 weeks from the transgene induction	70%	^104^
*UHRF1‐GFP*	*fabp10*	80% of *UHRF1‐GFP* transgenic zebrafish die by 20 dpf. Among the fish survived for more than 15 dpf, 76% of them develop HCC by 20 dpf	20 dpf	76%	^111^
*Twist1‐ERT2*	*fabp10*	80% of *Twist1‐ERT2/xmrk* double‐transgenic zebrafish shows spontaneous dissemination of mCherry‐labeled hepatocytes from the liver to the entire abdomen region and the tail region within 5 days from Twist1‐ERT2 activation	5 days from Twist1‐ERT2 activation	80%	^115^

### 4.1 HBx‐driven HCC models

Large numbers of clonally integrated HBV genomes, which were harbored by HCC and transgenic mice expressing HBx, developed HCC spontaneously.^19^, ^20^, ^22^ In an *HBx* transgenic zebrafish model, a fusion protein of HBx and mCherry (HBx‐mCherry) was expressed under the control of the liver‐specific *fabp10* promoter. Hematoxylin and eosin (H&E) staining analysis revealed that, in wild‐type (WT) background specimens, the zebrafish resulted in hyperplasia (10%), dysplasia (5%), or steatosis (40%) at 11 months postfertilization (mpf); however, in *TP53* null mutation background (*TP53^−/−^*), the zebrafish developed HCC (44%), dysplasia (17%), or steatosis (11%) at 11 mpf. Immunohistochemistry and quantitative RT‐PCR analyses showed that 29% of the zebrafish in *TP53^−/−^* had potent levels of nuclear PCNA accumulation in the liver and cell cycle‐related genes such as *ccna1*, *ccnb1*, *ccng1*, *cdk1*, and *cdk2* were dramatically upregulated; conversely, none of the zebrafish in WT exhibited these levels nor the elevated expression of these genes. In hyperplasia and HCC derived from the zebrafish in *TP53^−/−^*, high Src expression and elevated phosphorylation levels of ERK and Akt were observed. In contrast, in the livers derived from the zebrafish in WT and *TP53^−/−^*, Src was not detected. By crossing *HBx‐mCherry* with *Src* transgenic zebrafish line that expresses Src under control of the liver‐specific *fabp10* promoter, *HBx‐mCherry* and *Src* double‐transgenic zebrafish were generated and 50% of the double‐line developed HCC in presence of TP53 by 14 mpf.^80^

These experimental data suggest that HBx‐mCherry itself could not confer malignant transformation on hepatic cells, but it may induce HCC development in cooperation with either *TP53^−/−^* background or Src‐mediated signalings. As HBx could compromise DNA damage checkpoints through binding and inhibiting TP53, HBx‐mCherry fusion protein might fail to interact with TP53.^81^ Therefore, the transgenic zebrafish require a *p53^−/‐^* condition to develop HCC.

### 4.2 HCV core protein‐driven HCC and ICC models

Clinical studies have indicated a strong association between HCV infection and HCC development.^24^ Moreover, HCV‐ and HCV‐related cirrhoses are also known to be risk factors for ICC.^82^

To date, two types of HCV core protein (HCP) transgenic zebrafish lines have been established. One is to constitutively express HCP under the hepatocyte‐specific promoter, *fabp10*. This transgenic line develops HCC only in combination with thioacetamide (TAA) treatment, which is known to induce fibrosis and cirrhosis in animal models. Histological analysis has revealed that livers from the HCP transgenic zebrafish alone do not show any pathologic changes; normal cellular structures with well‐preserved cytoplasm, nuclei, and nucleoli are well maintained. However, in the presence of TAA, the transgenic zebrafish develop HCC by 6 weeks. At 1 week of TAA treatment, the transgenic zebrafish show higher steatosis than control WT zebrafish. By 4 weeks of TAA treatment, the transgenic zebrafish show bile duct damage and, by 6 weeks of TAA treatment, the transgenic zebrafish showed nodules comprising highly differentiated HCC with trabecular structures. In contrast, similarly treated WT zebrafish show nodules only at more than 12 weeks of TAA treatment.^83^

The other transgenic line is to co‐express HCP and HBx under the same *fabp10a* promoter by using a doxycycline repressible system. Interestingly, HCP‐HBx double transgenic lines develop ICC instead of HCC. At 1 month of induction of HCP‐HBx expression, several morphological abnormalities in the liver were observed, including cytoplasmic vacuolation, bile duct dilation, and formation of fibrosis in 25% of the fish. The percentage of fibrosis fish increased to 45% at 3 months and 35% of the zebrafish showed early and severe ICC. In contrast, HCP and HBx single transgenic zebrafish did not exhibit any abnormalities or fibrosis in the liver. Transcriptomic analysis revealed that a set of genes related to cytoskeletal remodeling, cell adhesion, development, and cell cycle regulation were highly expressed in ICC from HCP‐HBx zebrafish. In particular, intense signals of TGF‐β1, phosphorylated‐p38, phosphorylated‐pERK1/2, and Smad3L were observed in neoplastic bile duct epithelial cells from HCP‐HBx zebrafish, whereas they were not detectable in the livers from HCP or HBx single transgenic zebrafish. Remarkably, knockdown of TGF‐β1 via injecting *tgfb1* morpholinos into abdomens of 2‐month‐old HCP‐HBx zebrafish reduced bile duct proliferation from 53% to 32%, fibrosis formation from 53% to 23%, and ICC development from 29% to 11%.^84^

These experimental data suggest that HCP itself could not confer malignant transformation on hepatic and biliary epithelial cells, but it may accelerate HCC and ICC development in cooperated with other carcinogens (eg, TAA) and oncogenes (eg, HBx).

### 4.3 β‐Catenin‐driven HCC model

Aberrant regulation of Wnt/β‐catenin signaling has been frequently observed in HCC.^43^, ^56^, ^57^, ^58^ To date, three types of transgenic zebrafish lines that show aberrant regulation of Wnt/β‐catenin signaling have been reported. One line had a heterozygous mutation in the *APC* gene (*APC^+/−^*) and the mutant allele expressed truncated APC. Spontaneous liver tumors developed in 17.6% (six out of 34) of aged *APC^+/−^* fish (>15 months). Hepatic adenomas showed an accumulation of nuclear β‐catenin, which is the hallmark of activated Wnt signaling, and a high degree of proliferation. An experiment using chemical carcinogen 7,12‐ dimethylbenz[a]anthracene (DMBA) showed that 70.8% of DMBA‐treated *APC^+/−^* fish developed neoplasms in the liver compared with 20.5% of DMBA‐treated WT fish.^85^

Another line expressed a mutated form of *Xenopus laevis ctnnb1/β‐catenin* under control of the hepatocyte‐specific promoter, fabp10. This transgene contained four point mutations that affected putative phosphorylation sites (S33A, S37A, T41A, and S45A); these mutations are frequently mutated in human HCC and generate constitutive active form of β‐catenin by interfering with its phosphorylation and subsequent degradation.^56^ The level of HCC in fish was 78% and 80% at 6 and 12 mpf, respectively. Cross‐species comparison of HCC derived from the fish and human HCC revealed an upregulation of several Wnt target genes (*myca*, *lef1*, *pparda*, and *sp5*), and striking transcriptional similarities were observed between liver tumors in the fish and human HCC. In vivo drug screenings of SP600125 and EMD 420123 fish identifications, both of which target c‐Jun N‐terminal kinase (JNK), have a suppressor effect on fish liver enlargement. Activated β‐catenin was related with hyperactivation of JNK signaling pathway in both zebrafish and human HCC. Inhibition of JNK decreased liver size, specifically in HCC cells expressing constitutive active form of β‐catenin. The β‐catenin‐specific growth inhibitory effect of targeting JNK was conserved in human liver cancer cells. The suppressor effects of SP600125 were confirmed in a mouse model of HCC.^86^

The other line expressed human CTNNB1^mt^ and zebrafish tcf7l2 in hepatocytes. By crossing *Tg(TRE:CTNNB1^mt^‐P2A‐tcf7l2)* with *Tg(fabp10a:TetON;TRE:eGFP‐kras^v12^)*, the double transgenic larvae showed significant hepatomegaly within 3 days from Dox treatment. Although KRAS activation resulted in lipid droplet accumulation and steatosis in the liver of the fish, Wnt and *Myc* activities significantly attenuated the accumulation and cell senescence triggered by KRAS^v12^ expression. In vivo drug screenings using the fish identified that MK8245 (SCD inhibitor) suppresses hepatomegaly in the fish; MK8245 also suppresses the proliferation of human HCC cells.^87^

Zebrafish models of β‐catenin‐driven HCC demonstrate that activation of β‐catenin promotes liver overgrowth. That is consistent with mouse models where its activation leads to increased hepatocyte proliferation and liver. Furthermore, MK8245 and SP600125, which are identified through in vivo drug screening using the models, suppress the proliferation of human HCC. These findings suggest that new insights from the models would lead to potential therapeutic strategies for targeting HCC.

### 4.4 *Xmrk* (EGFR)‐driven HCC model

**FIGURE 1:**
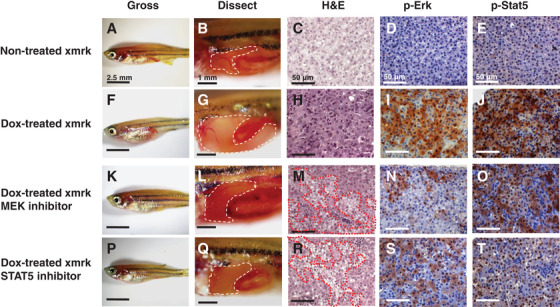
Effects of inhibition of p‐Erk and p‐Stat5 on HCC formation. (A‐E) Nontreated xmrk transgenic zebrafish as control. (F‐J) Doxycycline (Dox)‐treated xmrk transgenic zebrafish. (K‐O) Xmrk transgenic zebrafish treated with Dox and PD98059. (P‐T) Xmrk transgenic zebrafish treated with Dox and Stat5 inhibitor. Gross images of zebrafish, dissection to expose the liver, H&E staining, and IHC staining of phosphorylated Erk and Stat5 are indicated. Images are reprinted from ref. ^14^

Overexpression of EGFR is observed in 53% of the HCC patients.^75^ A hyperactive form of EGFR homolog in fish of the genus *Xiphophorus*, *Xmrk*, induces receptor dimerization and constitutive activation of downstream signaling in a ligand‐independent manner.^88^ Transgenic expression of *Xmrk* in the liver of zebrafish using a doxycycline (Dox) inducible system leads to HCC in essentially all juvenile fish within 3 weeks. All of the induced *Xmrk* transgenic zebrafish show HCC features,^13^ including disrupted two‐cell sheet organization, variation in nuclear and cellular sizes, syncytial cells, and vesicular nuclei with prominent and multiple nucleoli (Figure 1).^14^

An essential role in maintaining HCC in the zebrafish is *Xmrk*‐mediated signaling. Loss of *Xmrk*‐mediated signaling through the withdrawal of Dox regressed HCC in the induced transgenic zebrafish developing HCC. Histological examination revealed that neoplastic and normal hepatocytes coexist in the liver at 1 week from the Dox withdrawal, and the HCC features disappear by 2 weeks of Dox withdrawal. By 4 weeks of Dox withdrawal, all of the induced *Xmrk* transgenic zebrafish show normal liver morphology and histology. Moreover, resuming Dox treatment in HCC‐regressed transgenic zebrafish re‐induces the formation of HCC. Thus, the inducible *Xmrk* transgenic zebrafish model displays a clear oncogene‐addicted tumor formation.^14^

*Xmrk*‐mediated signaling contributes to the proliferation of hepatocytes in the transgenic zebrafish as immunohistochemical staining has revealed a dramatic increase in PCNA‐positive cells in the liver after Dox induction. In the process of HCC regression, the number of PCNA‐positive hepatic cells rapidly drops, whereas the number of apoptotic cells increases dramatically. In HCC developed in the induced *Xmrk* transgenic zebrafish, activation of ERK and STAT5 is also observed. Pharmacological blocking with either MEK/ERK or STAT5 inhibitors for 3 weeks suppresses HCC progression; these treatments lead to smaller abdomen and liver size. Histological analysis reveals that in the liver of either inhibitor‐treated zebrafish, normal and malignant hepatocytes coexist (Figure 1).^14^

These observations suggest that EGFR‐mediated signaling plays an essential role in not only HCC development but also in HCC maintenance. As clinical studies have indicated constitutive activation of Ras/ERK and Jak/STAT signaling in all cases of the clinical HCC samples analyzed (n > 80),^89^ the inhibitors tested in the *Xmrk* transgenic zebrafish may also be capable of regressing human HCC.

### 4.5 KRAS‐driven HCC model

In clinical studies, approximately 3% (1/30 cases) of HCC patients possessed constitutively active mutation of KRAS in which codon 12 of KRAS is mutated from glycine to valine and constitutive activation of Ras/ERK signaling is observed in all cases of over 80 clinical samples.^89^, ^90^ In experimental studies, it has been demonstrated that the HCV core protein activates MAPK/ERK signaling and blocking Raf/ERK signaling with sorafenib prevents PLC∖PRF∖5 and human HepG2 cells from proliferating in vitro and in vivo.^91^, ^92^ Therefore, KRAS‐mediated signaling plays a critical role in developing and maintaining HCC. Using a mifepristone‐inducible transgenic system, an EGFP fusion protein with KRAS^G12V^ (EGFP‐Kras^G12V^) has been expressed in a liver‐specific manner. All of *EGFP‐KRAS^G12V^* transgenic zebrafish developed robust and homogeneous tumors in the liver after 1 month of mifepristone induction.^93^

*EGFP‐KRAS^G12V^* expression in the liver induces malignant transform of hepatocytes through activating both Ras/ERK and PI3K/Akt pathways. Western blot and immunohistochemistry analyses have revealed that HCC derived from mifepristone‐treated KRAS transgenic zebrafish show activation of Ras/ERK and PI3K/Akt signaling pathways, and these active states are dramatically decreased after the withdrawal of mifepristone. The pharmacological block of Ras/ERK signaling with PD98059 (MEK inhibitor) suppresses hyperplastic liver growth in 49% of the zebrafish at the larval stage. Similarly, blocking PI3K/Akt/mTOR signaling with either LY294002 (PI3K inhibitor) or Rapamycin (mTOR inhibitor) inhibits the growth in over half of the larvae. Moreover, the combined treatment of PD98059 with either LY294002 or Rapamycin dramatically decreases the frequency of tumorigenesis.^13^ These observations suggest that Ras/ERK and PI3K/Akt/mTOR signaling pathways might play essential roles in not only developing but also maintaining HCC. Thus, pharmacological block with multiple signaling pathways could be promising and effective for eliminating human HCC.

### 4.6 *Myc*‐driven HCC model

Chromosomal amplification at 8q, where the *Myc* gene is located, is observed in 48% (24/50 cases) of HCC patients and elevated expression of *Myc*‐regulated genes is strongly associated with a malignant conversion of HCC.^94^, ^95^ Therefore, *Myc* is thought to be an oncogenic driver in hepatocarcinogenesis. To date, two types of *Myc* transgenic zebrafish have been established: one expresses mouse *Myc* in a liver‐specific manner using a doxycycline‐inducible system and the other expresses *EGFP*‐fused gene with either zebrafish *myca* or *mycb* in the liver by using a mifepristone‐inducible system.^96^

The induced liver expression of the mouse *Myc* in transgenic zebrafish causes liver hyperplasia, which is progressed to hepatocellular adenoma and carcinoma with prolonged transgene expression. Transcriptomic analysis reveals that upregulated genes in *Myc*‐driven zebrafish liver tumors are similar to those in human liver tumors. In particular, the ribosome biogenesis constitutes the key features of *Myc*‐driven carcinogenesis in the liver.^96^

Transgenic zebrafish with *myca* expression show stronger phenotype than *mycb* transgenic zebrafish and develop different types of liver tumors. At 1 month from the transgene induction, 87% of the *myca* transgenic zebrafish show ascites like phenotype with yellow fluid in the abdomen cavity as well as fluid‐filled cysts in the liver. At 6‐7 months from the transgene induction, 51% of *myca* fish develop large tumors, 14% show large tumors with overt angiogenesis, and 7% retain ascites‐like phenotype with a pseudoglandular organization of tumor cells. At 8‐9 months from the transgene induction, about 5% of the induced fish develop multinodular HCC with cirrhosis. In this multinodular HCC, loss of E‐cadherin and β‐catenin accumulation in the nuclear compartment is observed. Furthermore, loss of the transgene through mifepristone withdrawal resulted in tumor regression through inducing apoptosis.^52^

These observations suggest that *myca* plays an essential role in not only HCC development but also in HCC maintenance. Clinical studies show that elevated expression of *Myc*‐regulated genes is strongly associated with a malignant conversion of HCC.^94^, ^95^ Therefore, identifying the *Myc*‐regulated genes that play an essential role in maintaining HCC growth of the transgenic fish would lead to potential therapeutic strategies for targeting HCC.

### 4.7 Endothelin 1‐driven HCC model

Endothelin 1 (*EDN1*) is a potent vasoconstrictor peptide, which is composed of 21 amino acids. The biological effects of endothelins are mediated by plasma membrane‐bound receptors (ETA and ETB). Apart from being a recognized vasoconstrictor peptide, clinical studies reported that plasma concentration of *EDN1* was markedly increased in both HCC patients and patients with metastasized tumors in the liver and that 70% (n = 14/20) of HCC samples showed *EDN1* expression.^97^, ^98^ In a rat hepatoma model, it has been demonstrated that exogenous addition of *EDN1* enhances hepatoma cell growth in a dose‐dependent manner, whereas an endothelin receptor antagonist inhibits tumor growth.^99^ Moreover, in a HBV X antigen‐induced HCC mouse model, *EDN1* is one of the genes that are induced by X antigen.^40^, ^100^ Therefore, *EDN1* is thought to be an oncogenic driver in hepatocarcinogenesis. Transgenic *EDN1* zebrafish that express human *EDN1* under control of the liver‐specific *fabp10a* promoter are established. H&E staining analysis revealed that 83% of the zebrafish showed steatosis by 5 months, 17‐18% of the zebrafish developed hyperplasia by 7‐9 months, and 17‐20% of the zebrafish exhibited HCC by 11 mpf. Quantitative RT‐PCR analysis showed that elevated expression of lipogenic factor and enzymes (*srebf1*, *cebpa*, *fasn*, and *adapt*) and significant upregulation of cell cycle‐related genes (*ccna1*, *ccnb1*, *ccne1*, *ccng1*, *cdk1*, and *cdk2*) were observed in the liver of the zebrafish at 5 and 11 mpf, respectively. Immunohistochemistry analyses showed that potent levels of nuclear PCNA accumulation and elevated phosphorylation level of AKT were detected in the liver of the zebrafish at 9 and 11 mpf; conversely, only low levels of PCNA and phosphorylation level of AKT were detected in the liver of control zebrafish at the same age.^101^

These experimental data suggest that *EDN1* might promote cell proliferation through activating PI3K/AKT signaling pathway, and that might lead to HCC development.

### 4.8 Transforming growth factor‐β‐driven HCC model

The pleiotropic cytokine transforming growth factor‐β (TGFβ) plays a bifunctional role in either inhibiting or promoting cell proliferation. During hepatocarcinogenesis, it acts as tumor suppressor and tumor promoter in the early and late stages, respectively.^102^ Clinical studies reveal that HCC patients show a distinct signature of TGF‐β gene expression and the signature correlates with tumor invasiveness, the time before relapse, and long‐term survival of the patient.^103^

An inducible *tgfb1a* transgenic zebrafish line that expresses zebrafish *tgfb1a* in the liver by using a mifepristone inducible system^96^ has been established. Depending on the intensity of TGFβ1 induction, the fish developed different lesions in the liver; low and moderate levels of TGFβ1 caused hyperplasia and hepatocellular adenoma and high levels of TGFβ1 caused HCC to develop. In the HCC cells, TGFβ1‐mediated signaling switched from Smad to ERK‐mediated signaling. Although pharmacological inhibition of Smad‐mediated signaling with PD169316 (Smad2 inhibitor) does not affect HCC development in the fish, ERK‐mediated signaling with U0126 (MEK inhibitor) suppresses the proliferation of hepatic cells.^104^

An inducible tgfb1a transgenic zebrafish demonstrated that overexpression of tgfb1a alone is sufficient to induce HCC. Clinical studies reveal that TGF‐β expression correlates with tumor invasiveness, the time before relapse, and long‐term survival of the patient with HCC^103^; and constitutive activation of ERK signaling is observed in all cases of over 80 clinical samples.^89^, ^90^ Therefore, pharmacological inhibition of ERK‐mediated signaling with U0126 (MEK inhibitor) may be effective for eliminating TGF‐β expressing HCC.

### 4.9 Ubiquitin‐like with PHD and RING finger domains 1‐driven HCC model

Ubiquitin‐like with PHD and RING finger domains 1 (UHRF1) is a member of a subfamily of RING‐finger‐type E3 ubiquitin ligases, consisting of multiple domains: a N‐terminal ubiquitin‐like domain; tandem tudor and PHD domains for recognizing methylated histones; an SRA domain for interacting with hemimethylated DNA, DNA methyltransferase 1 (DNMT1), and histone deacetylase 1 (HDAC1); and a C‐terminal RING finger motif for E3‐ubiquitin ligase activity.^105^ It contributes to not only cell cycle regulation and epigenetic modifications via recruiting DNMT1 and HDAC1, but also to TP53‐dependent DNA damage checkpoints.^106^ In primary cultured human lung fibroblasts, UHRF1 expression peaked at late G1 and during the G2/M phase; conversely, in cancer cell lines such as HeLa, Jurkat, and A549, constant UHRF1 expression is observed throughout the entire cell cycle.^107^ In clinical studies, elevated expression of UHRF1 is observed in several tumors from breast, lung, bladder, pancreas, colon, and prostate.^108^, ^109^, ^110^ Therefore, UHRF1 is thought to be an oncogenic driver in tumorigenesis.

Recently, a transgenic zebrafish line that expresses the human *UHRF1* gene fused to GFP (*UHRF1‐GFP*) in hepatocytes to induce HCC has been reported. In WT background, 74% of larval UHRF1‐GFP transgenic zebrafish have small livers and 80% of them die by 20 days postfertilization (dpf). In the small zebrafish livers at 5 dpf, intense senescence‐associated β‐galactosidase (SA‐β‐gal) staining is detected. Among the *UHRF1* transgenic zebrafish survived for more than 15 dpf, 76% of them develop HCC by 20 dpf. Moreover, HCC incidence at 15 dpf in *UHRF1* transgenic fish increased from 46% in the WT background to 87% in *TP53* heterozygous mutation background.^111^ These observations suggest that a bypass of TP53‐induced senescence would be required for *UHRF1* to act as an oncogene.

Furthermore, clinical samples from 58 patients with dysplastic nodules (n = 18) or HCC (n = 40) have shown an average of 20‐ and 40‐fold overexpression of *UHRF1* in advanced and very advanced HCC cases. Knockdown of *UHRF1* by RNA interference in the human hepatic cancer cell line, HepG2, induces PARP cleavage and other markers of apoptosis.^111^ Therefore, therapeutic strategies targeting UHRF1 might regress human HCC.

### 4.10 Twist1‐induced metastasis models

HCC is a rapidly growing tumor associated with a high propensity for vascular invasion and metastasis. Epithelial‐mesenchymal transition (EMT), which converts various types of epithelial cells into mesenchymal cells, plays a critical role in promoting metastasis.^112^ Twist is an EMT‐inducible transcriptional factor; elevated expression of Twist is associated with poor survival rates in cancer patients.^113^ Immunohistochemical microarray stainings of paired primary and metastatic HCC cells revealed that overexpression of Twist was correlated with HCC metastasis.^114^

**FIGURE 2:**
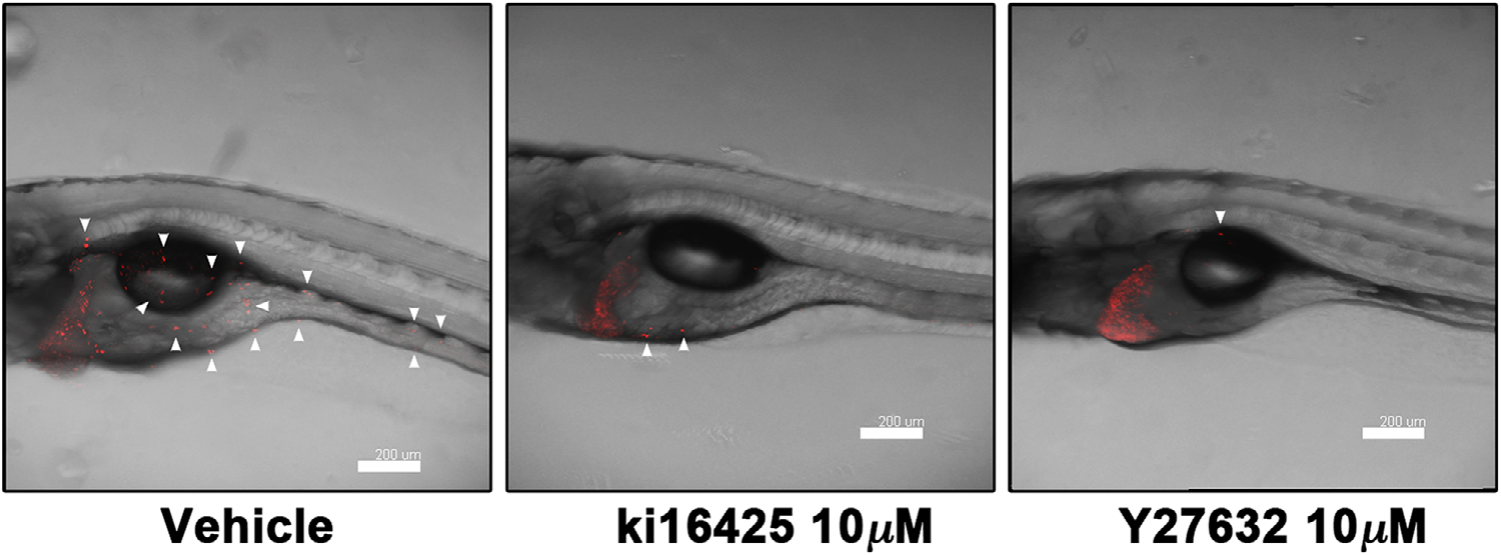
Reported antimetastasis drugs: ki16425 and Y27632 could suppress dissemination of mCherry‐labeled hepatic cells from the liver of *Twist1a‐ER^T2^/xmrk* double transgenic zebrafish. Representative images of the dissemination in the fish that were treated with doxycycline and 4‐OHT in presence of vehicle (left), 10 mmol/L of ki16425 (middle), or 10 mmol/L of Y27632 (right). Some disseminated mCherry‐positive cells are indicated by arrowheads. The images were shown as Z‐stack images using 100× magnification. Scale bar, 200 μm. Images are reprinted from ref. ^115^

A tamoxifen‐controllable Twist1a‐ER^T2^ transgenic zebrafish is reported to model metastatic dissemination of hepatic cells. The activation of Twist1a‐ER^T2^ following 48 h of tamoxifen treatment induces EMT in the liver. By crossing this model with *Xmrk* transgenic zebrafish, approximately 80% of the double‐transgenic zebrafish show spontaneous dissemination of mCherry‐labeled hepatocytes from the liver within 5 days from the treatment initiation. This rapid and high‐frequency induction of the dissemination provides a novel way to screen chemicals/drugs in vivo for identification of antimetastasis drugs targeting metastatic dissemination of cancer cells (Figure 2).^115^

In vivo drug screening using *Twist1a‐ER^T2^/Xmrk* double transgenic zebrafish identifies that adrenosterone, which inhibits hydroxysteroid (11‐beta) dehydrogenase 1 (HSD11β1), suppresses dissemination of mCherry‐labeled hepatic cells from the liver of *Twist1a‐ER^T2^/xmrk* double transgenic zebrafish. This suppressor effects is validated in a zebrafish xenotransplantation model where highly metastatic human HCC cell line HCCLM3 and breast cancer cell line MDA‐MB‐231 are inoculated into the duct of Cuvier of *Tg (kdrl:EGFP)* transgenic zebrafish. Pharmacological and genetic inhibition of HSD11β1 induces the re‐expression of E‐cadherin and other epithelial markers and the lost partial expression of mesenchymal markers via the downregulation of Snail and Slug.^115^

These observations suggest, in this model, Twist1a‐ER^T2^‐driven EMT alone would not be sufficient to induce abdominal and distant cell dissemination and that cooperation of *Xmrk*‐driven cellular events would be required for dissemination.

## 5 INSIGHTS FROM ZEBRAFISH MODELS OF HCC FOR DEVISING NOVEL THERAPEUTIC STRATEGIES FOR HCC

The HCC tumor is complex and heterogeneous and generally has multiple genomic alterations. These alterations lead to the aberrant activation of cellular kinase‐signaling networks to confer malignant transformation and proliferation of cancer cells. Targeting a single kinase has proven successful in some cancer cases; for example, Imatinib (Gleevec, Novartis) eliminates Philadelphia chromosome‐positive B cell acute lymphoblastic leukemia by inhibiting BCR‐ABL and gefitinib (Iressa, AstraZeneca) inhibits the progression of nonsmall lung cancer by interrupting EGFR tyrosine kinase activity.^116^, ^117^ However, this approach has faced difficulties because cancer cells acquire resistance to these kinase inhibitors.^118^ Recently, sorafenib (BAY43‐9006; Nexavar), a multikinase inhibitor, has been shown to have survival benefits in patients suffering from renal, melanoma, or HCC^119^ cancers. To overcome the complexity of aberrant activation of the HCC signaling network, combination therapies that target multiple signaling pathways would be promising and effective.

### 5.1 Sorafenib and rapamycin combination therapy

Sorafenib is a kinase inhibitor targeting Raf kinase and several receptor tyrosine kinases, including platelet‐derived growth factor receptor (PDGFR), vascular endothelial growth factor receptor 2 (VEGFR2), FLT3, c‐Kit, and Ret. Sorafenib has been considered the standard of care for patients with advanced HCC since 2007.^119^, ^120^ However, a phase II trial involving 137 patients with advanced HCC showed that sorafenib treatment resulted in a median overall survival of 9.2.^121^ This suggests that a single sorafenib treatment resulted in modest efficiency. A combination therapy of sorafenib with other kinase inhibitors could be more effective.

Activation of Ras/ERK signaling has been observed in 100% of over 80 clinical HCC samples.^89^ Aberrant mTOR signaling (p‐RPS6) is present in half of HCC specimens (n = 314).^122^ In a *KRAS^G12V^* transgenic zebrafish model, it has been shown that pharmacological blocking of Ras/MEK and PI3K/mTOR signalings with PD98059 (MEK inhibitor) and rapamycin (mTOR inhibitor) suppresses the enlargement of oncogenic livers.^13^ Thus, we propose that a combination therapy of sorafenib with rapamycin may have a better effect in HCC treatment. An experimental study has demonstrated that the combination therapy strongly inhibits primary tumor growth and lung metastases in a mouse model xenografted with human HCC cells (LCI‐D20); the inhibition is resultant of therapy‐induced apoptosis and suppressed angiogenesis.^123^

### 5.2 Combination therapy using gefitinib and ruxolitinib

Gefitinib is an EGFR inhibitor that interrupts EGFR‐mediated Ras/MEK, PI3K/AKT, and Jak/STAT signaling pathways by binding to the ATP‐binding site of EGFR tyrosine kinase. Blockading EGFR signaling with gefitinib prevents DEN‐induced liver carcinogenesis in rats and inhibits the proliferation of human HCC cell lines Huh‐7 and HepG2.^78^, ^79^ Despite these findings, a phase II study of erlotinib, which has a similar chemical compound as gefitinib, showed poor effects in regressing HCC (n = 38).^124^

It has been reported that nonsmall lung cancer cells acquire resistance to gefitinib via bypassing downstream signaling of EGFR.^125^ In our *Xmrk* transgenic zebrafish, we have demonstrated that the loss of EGFR‐mediated signaling regresses liver tumors and pharmacological blocking with MEK and STAT5 inhibitors also interrupts liver tumor formation (Figure 1).^14^ These observations suggest that double blocking Ras/ERK and Jak/STAT signalings would be more effective in inhibiting the proliferation of HCC cells. We therefore propose a combination HCC therapy of gefitinib with an inhibitor that targets Jak/STAT signaling. Fortunately, the Jak1/2 inhibitor ruxolitinib (INC424; Novartis) has already been approved by the FDA for the treatment of intermediate or high‐risk myelofibrosis, and an experimental study has demonstrated that ruxolitinib has an antiproliferative effect on HCC.^126^ Therefore, a drug repositioning of ruxolitinib would be desired for the combination therapy.

## 6 CONCLUSION

**FIGURE 3:**
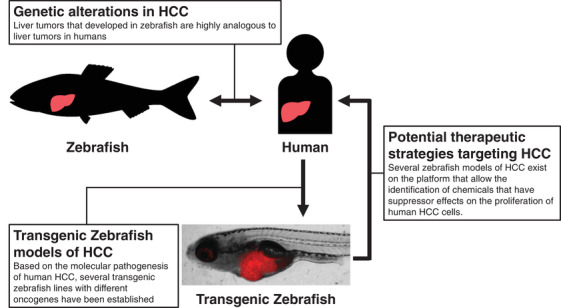
Liver tumors that developed in zebrafish as a result of exposure to chemical carcinogens are highly analogous to liver tumors in humans. Based on the molecular pathogenesis of human HCC, several transgenic zebrafish lines with different oncogenes have been established. Some of them exist on the platform that allow the identification of chemicals that have suppressor effects on the proliferation of human HCC cells. Image is reprinted from ref. ^115^

The zebrafish modeling system has been increasingly recognized as a platform for chemical screening because it provides the advantage of high‐throughput screening in an in vivo vertebrate setting with physiologic relevance to humans.^6^, ^7^, ^8^, ^9^, ^10^ Our early comparative transcriptomic analysis demonstrated that liver tumors that developed in zebrafish as a result of exposure to chemical carcinogens are highly analogous to liver tumors in humans.^15^ Based on the molecular pathogenesis of human HCC, several transgenic zebrafish lines with different oncogenes have been established. Some of them exist on the platform that allow the identification of chemicals that have suppressor effects on the proliferation of human HCC cells (Figure 3). A few chemicals identified in zebrafish screening have reached clinical trials.^127^, ^128^, ^129^ Therefore, there is the possibility that the chemicals identified by zebrafish HCC modeling might reach clinical trials. Furthermore, studies using *Xmrk* and *myca* transgenic zebrafish demonstrate that EGFR‐mediated signaling and *Myc*‐regulated genes play an essential role not only in HCC development but also in HCC maintenance. Therefore, identifying the EGFR‐mediated signaling and the *Myc*‐regulated genes that play an essential role in maintaining HCC growth of these fish could lead to potential therapeutic strategies for targeting HCC.

## CONFLICT OF INTEREST

The authors declare no conflict of interest.
